# Non-typhoidal salmonella: an unusual cause of spontaneous bacterial peritonitis in decompensated cirrhosis

**DOI:** 10.1093/gastro/gou018

**Published:** 2014-03-19

**Authors:** Tony Joseph, Prasanth Sobhan, Suthanu Bahuleyan, Anil John, Shanid Abdul Sathar, Srijaya Sreesh, Kattoor Ramakrishnan Vinayakumar

**Affiliations:** Department of Medical Gastroenterology, Government Medical College, Thiruvananthapuram, Kerala, India

**Keywords:** spontaneous bacterial peritonitis, salmonella typhimurium, ascitic fluid protein

## Abstract

*Salmonella typhimurium*, a non-typhoidal salmonella, is an unusual cause of spontaneous bacterial peritonitis (SBP). It is usually reported in asymptomatic patients with normal or high ascitic fluid protein levels with underlying immunosuppression, as high opsonic activity in the ascitic fluid of these patients protects them from the usual organisms causing spontaneous bacterial peritonitis, unless they are exposed to a particularly virulent organism like salmonella. We report a case of culture-proven non-typhoidal salmonella in a patient with decompensated cirrhosis, with low protein and without any underlying immunosuppression, and no other source to explain its origin.

## INTRODUCTION

Spontaneous bacterial peritonitis (SBP) is the most frequent bacterial infection in cirrhosis, estimated to occur in 10–30% patients with cirrhosis [1]. It requires prompt diagnosis because it carries a mortality of around 30% [2]. Among those surviving the first episode, 50–70% carry a risk of further episodes within one year [3]. Bacterial translocation has been recognised as the major mechanism of SBP [4]. The micro-organisms usually isolated from cases of SBP are those that are able to translocate into the mesenteric lymph node, such as *E. coli*, *K. pneumoniae* and other enterobacteriaceae [5]. We report a case of SBP due to *S. typhimurium* in a patient with decompensated cirrhosis.

## CASE REPORT

A 57-year-old female presented with history of progressive painless abdominal distension of 10 days duration. Historically, there was no fever, pedal oedema, orthopnoea, facial puffiness, decreased urine output, altered sensorium, altered bowel habits or overt gastro-intestinal bleeding manifestations. She had a history of gradual weight loss totalling 13 kg over the previous 5 years. She had been diabetic for three years and was presently on oral hypoglycemics. She was diagnosed to have sputum-positive pulmonary tuberculosis eight months back and was treated with isoniazid (H), rifampicin (R), pyrazinamide (Z) and ethambutol (E) during the intensive phase for 2 months and with H and R for 3 months. Two months back, while on the continuation phase of anti-tubercular drugs, she was hospitalised with jaundice. Anti-tubercular drugs were stopped, in view of raised bilirubin and transaminases. Review of her old medical records during the episode revealed the results of liver function tests as follows: bilirubin total/direct (Bil.T/D) = 17.6/7.1 mg/dL, aspartate/alanine transferase (AST/ALT) = 1577/1600 IU/L, alkaline phosphatise (ALP) = 157 U/L, total protein/albumin (TP/Alb) = 7.8/3.6 g/dL, prothrombin time (PT) = 20 s (control 13.4 s) and international normalised ratio (INR) = 1.79. On discharge these figures were: Bil.T/D = 15.6/13.1, AST/ALT = 173/175, ALP = 150, TP/Alb = 6.1/2.2, PT = 22.2 and INR = 1.9. Ultrasound sonography evaluation during that admission showed evidence of chronic liver disease and portal hypertension. Ascites was absent. She was managed conservatively; serial monitoring of liver function tests was done and she was subject to regular follow- up. She had history of postpartum sterilisation, carried out 25 years back. There was no history of gall bladder symptoms. There was no history of chronic liver disease or gastro-intestinal malignancy in the family. There was history of diabetes, hypertension and dyslipedemia in the family.

On examination her vital signs were stable. Her weight was 64 kg; she was icteric and also had pedal edema. Gastro-intestinal system examination revealed marginally palpable liver, enlarged spleen palpable 2 cm below the left costal margin and shifting dullness. There was no asterixis. Other systems were normal. Her haemogram, renal function and electrolytes were normal. Abnormal tests were HbA1c 12.1%; liver function tests showed Bil.T/D = 10/3.3, AST/ALT = 115/71, ALP = 117, TP/Alb = 6.6/2.1, PT = 18.4 and INR = 1.53. Ascitic fluid study demonstrated protein of 1 g%, Alb 0.5 g% with serum ascites albumin gradient (SAAG) of 1.6, consistent with portal hypertension. Ascitic fluid absolute neutrophil count was 618 and lactate dehydrogenase (LDH) 366. Ultrasound sonography showed coarsened liver echotexture with irregular surface, enlarged spleen and ascites. There was no portal vein thrombosis or focal liver lesions. Gall bladder was normal. Upper endoscopy showed Grade II varices without red colour sign.

**Figure 1.**
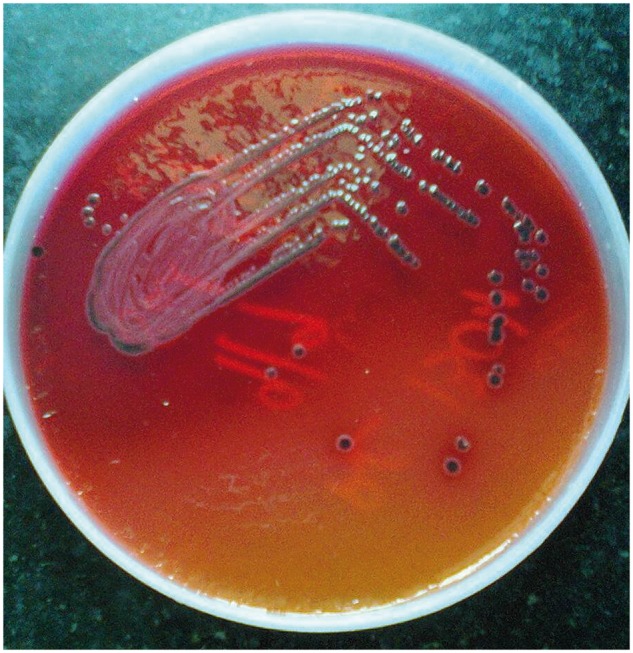
Ascitic fluid culture showed positive for *Salmonella typhimurium* in Xylose-Lysine Deoxycholate Agar.

A diagnosis of chronic liver disease with present decompensation precipitated by acute insult in the form of drug-induced liver injury was considered. Aetiologies considered were occult hepatitis due to hepatitis B, hepatitis C and non-alcoholic steatohepatitis causing chronic liver disease, in view of her diabetes and obesity in the past. Ascitic fluid culture was positive for *S. typhimurium* in xylose-lysine deoxycholate agar (XLD agar) (Figure 1). Widal test and Enterocheck® test for salmonella were negative. Blood, sputum, stool and urine cultures were sterile. Sputum acid-fast bacilli (AFB) test was negative. Chest X-ray (postero-anterior) was normal. Her viral serology was negative for hepatitis B, C and human immunodeficiency virus. AntiHbc, HBV DNA PCR and HCV RNA PCR were all negative. Serum ceruloplasmin and antinuclear antibody was also negative.

The patient was diagnosed as having chronic liver disease with portal hypertension, with etiology probably related to non-alcoholic steatohepatitis (NASH), with present decompensation due to drug-induced liver injury and SBP due to *S. typhimurium*. She was treated with empirical, parenteral therapy with cefotaxime (2 g t.i.d) for 5 days. Ascitic fluid study done after 48 hours showed >25% reduction in absolute neutrophil count. The patient had an uneventful course during hospitalisation and completely recovered, as repeat culture after five days of cefotaxime therapy was sterile.

## DISCUSSION

Salmonella typhimurium is a pathogenic gram-negative bacteria predominately found in the intestinal lumen. It causes gastroenteritis in humans and other mammals. In mice it causes symptoms resembling those of typhoid fever in humans [6]. It is a rare cause of SBP. The antimicrobial (opsonic) activity in ascitic fluid depends on the protein content of the ascitic fluid, the level of immune defence of the host and the virulence of the organism. Low-protein ascitic fluids in cirrhotic patients are deficient in opsonic activity and are particularly predisposed to spontaneous bacterial peritonitis. It is thought that patients with normal or high protein levels appear to be protected from spontaneous bacterial peritonitis unless they are exposed to a particularly virulent organism such as salmonella [7].

In previous case reports, an underlying immunocompromised state, such as AIDS, extrahepatic malignancies and chemotherapy were the predisposing factors for SBP due to *salmonella spp*. Those cases were mostly asymptomatic and had relatively normal ascitic fluid protein levels. It is possible that an underlying immunosuppressed state prevented from mounting an immunological response [8–11].

In our patient, the *S. typhimurium*, a non-typhoidal salmonella, might have translocated from the gut, since she was asymptomatic and there was no other source to explain its origin. The presence of uncontrolled diabetes could be an additional predisposing factor in our patient.

In summary, normal or high protein levels should not be considered a protective factor for spontaneous bacterial infection by non-typhoidal salmonella, as cited in the literature as well as previous case reports. It may be the bacterial virulence that is the most important. Non-typhoidal salmonella SBP should be considered, even in the absence of underlying immunosuppression. Since the empirical treatment with cefotaxime seems to be the most efficient (as it is very active *in vitro* against salmonella and also against other common enterobacteriaceae implicated in SBP), it is the ascitic fluid culture study that is the most important in identifying these unusual organism.

**Conflict of interest:** none declared.
